# Does Regulation 28 Prevent Future Deaths? A Scoping Review of Coroners’ Prevention of Future Death (PFD) Reports in Mental Health

**DOI:** 10.1192/bjo.2026.11100

**Published:** 2026-06-30

**Authors:** Tajnin Mitu, Asmaa Elsayed, Alberto Salmoiraghi, Heidi Hales

**Affiliations:** 1 Betsi Cadwaladr University Health Board, Wrexham, United Kingdom; 2 Betsi Cadwaldr University Health Board, Wrexham, United Kingdom

## Abstract

**Aims:**

Regulation 28 of the Coroners and Justice Act 2009 enables coroners in England and Wales to issue Prevention of Future Death (PFD) reports when systemic risks are identified. Organisations must respond within 56 days outlining actions taken. Although aimed to prevent future deaths, there is paucity of evidence in regard to the effectiveness of Regulation 28.

This review aimed to map published analyses of mental health- and suicide-related PFD reports, identify recurring systemic concerns, and assess whether Regulation 28 functions as an effective preventive mechanism.

**Methods:**

A scoping review was conducted using Arksey and O’Malley’s framework to synthesise heterogeneous evidence. Database used were Embase, Medline and Psychinfo (from 2010–2013) with search terms: Regulation 28; Mental Health; suicide; prevention of future death.

• Included studies were those that:

• Analysed Regulation 28 PFD reports.

• Focused on suicide, mental health-related deaths, medicines implicated in suicide, autism-related deaths, or organisational learning.

A narrative synthesis identified cross-cutting structural domains including communication, risk assessment, service access, prescribing, workforce capacity, and governance.

**Results:**

The search identified 8 studies to include in the scoping review.

Recurring themes were consistent across populations:

Communication failures – poor inter-agency information sharing and discharge planning.

Inadequate risk assessment – incomplete documentation and over-reliance on risk tools.

Barriers to access – long waits, rejected referrals, and crisis care gaps.

Staffing and training deficits – workforce shortages and limited suicide prevention training.

Medicines-related risks – opioids (40%) and antidepressants (30%) frequently implicated.

Policy non-compliance – failure to follow existing procedures.

Response rates to PFDs were inconsistent (as low as 58% in some analyses), and no enforcement mechanism exists. Similar concerns recur across years, suggesting limited demonstrable preventive impact.

**Conclusion:**

Regulation 28 consistently identifies modifiable systemic failings and functions effectively as a diagnostic learning tool. Whilst Regulation 28 holds preventive potential, the effectiveness remains unclear. Its preventive capacity is constrained by weak oversight, inconsistent accountability, and absence of outcome tracking. Without mandatory oversight, standardised reporting, and measurable outcome tracking, PFDs risk remaining a mere procedural issue rather than effectively preventing future deaths.

In summary, though Regulation 28 has theoretical preventive potential, stronger national coordination, mandatory implementation monitoring, and integration into regulatory frameworks are required to translate coronial learning into measurable reductions in mental health-related mortality.

